# Genome-wide identification, phylogeny and expression analysis of AP2/ERF transcription factors family in *Brachypodium distachyon*

**DOI:** 10.1186/s12864-016-2968-8

**Published:** 2016-08-15

**Authors:** Licao Cui, Kewei Feng, Mengxing Wang, Meng Wang, Pingchuan Deng, Weining Song, Xiaojun Nie

**Affiliations:** 1 State Key Laboratory of Crop Stress Biology in Arid Areas, College of Agronomy and Yangling Branch of China Wheat Improvement Center, Northwest A&F University, Yangling, 712100 Shaanxi China; 2 Australia-China Joint Research Centre for Abiotic and Biotic Stress Management in Agriculture, Horticulture and Forestry, Yangling, 712100 Shaanxi China

**Keywords:** Abiotic stress, AP2/ERF, Brachypodium, Expression profiles, Gene family, Transcription factor

## Abstract

**Background:**

The AP2/ERF transcription factor is one of the most important gene families in plants, which plays the vital role in regulating plant growth and development as well as in response to diverse stresses. Although AP2/ERFs have been thoroughly characterized in many plant species, little is known about this family in the model plant *Brachypodium distachyon*, especially those involved in the regulatory network of stress processes.

**Results:**

In this study, a comprehensive genome-wide search was performed to identify AP2/ERF gene family in Brachypodium and a total of 141 BdAP2/ERFs were obtained. Phylogenetic analysis classified them into four subfamilies, of which 112 belonged to ERF, four to RAV and 24 to AP2 as well as one to soloist subfamily respectively, which was in accordance with the number of AP2 domains and gene structure analysis. Chromosomal localization, gene structure, conserved protein motif and cis-regulatory elements as well as gene duplication events analysis were further performed to systematically investigate the evolutionary features of these BdAP2/ERF genes. Furthermore, the regulatory network between BdAP2/ERF and other genes were constructed using the orthology-based method, and 39 BdAP2/ERFs were found to be involved in the regulatory network and 517 network branches were identified. The expression profiles of BdAP2/ERF during development and under diverse stresses were investigated using the available RNA-seq and microarray data and ten tissue-specific and several stress-responsive BdAP2/ERF genes were identified. Finally, 11 AP2/ERF genes were selected to validate their expressions in different tissues and under different stress treatments using RT-PCR method and results verified that these AP2/ERFs were involved in various developmental and physiological processes.

**Conclusions:**

This study for the first time reported the characteristics of the BdAP2/ERF family, which will provide the invaluable information for further evolutionary and functional studies of AP2/ERF in Brachypodium, and also contribute to better understanding the molecular basis for development and stresses tolerance in this model species and beyond.

**Electronic supplementary material:**

The online version of this article (doi:10.1186/s12864-016-2968-8) contains supplementary material, which is available to authorized users.

## Background

Plant growth, development and productivity are adversely affected by numerous abiotic stresses, such as drought, salt and heat. To survive and flourish under these environmental stresses, plants have developed a complicated response mechanism by repressing or inducing the expression of a series of genes with diverse functions. Transcription factors (TF), as an important group of regulatory proteins, play the central roles in regulation network and signaling pathways of plant development and in response to abiotic stresses. Among them, AP2/ERF (APETALA2/Ethylene Responsive Factor) superfamily is one of the biggest plant TFs, which distinguished by one or two highly conserved ethylene-responsive element-binding factor domains that consisted of 50–60 amino acids [1, 2]. Based on sequence similarities and repetitions of AP2 DNA-binding domains, it can be classified into AP2, ERF and RAV families [3]. The members of AP2 family proteins contain two AP2/ERF domains and are further divided into AP2 and AINTEGUMENTA (ANT) monophyletic groups [4, 5], while the members of ERF subfamily possesses a AP2/ERF domain with the specific WLG motif and are subdivided into ten group [3], of which Group I to IV belong to the DREB subfamily and group V to X belong to the ERF subfamily. The ERF subfamily is characterized by an additional cis-acting element AGCCCGCC of the GCC-box in the promoter regions [6], whereas the DREB subfamily typically binds to dehydration-responsive element-binding factor, which has a core motif of CCGAC [7]. The RAV family members containing the single AP2/ERF domain and a specific B3 DNA-binding motif [8]. In addition, other members with an AP2-like domain but lacking additional motifs are often defined as Soloist.

Extensive studies have revealed the crucial role of the AP2/ERF genes playing in plant growth, development and stress responses [4, 9–11]. Generally, the AP2 subfamily members were the main factors involving in regulating organ architecture and development, such as leaf epidermal cell determinacy, spikelet meristem differentiation and floral organ patterning [12] as well as seed mass and seed yield [13, 14], while the RAV subfamily showed the important functions in plant hormone signal transduction, such as ethylene [15], Brassinosteroid [16], and also involved in response to biotic and aboitic stresses [17, 18]. Additionally, the DREB, together with other members in ERF subfamily mainly involved in response to biotic and abiotic stresses, such as water deficit [19], low and high temperature [20, 21] and high salinity [22].

*B. distachyon*, belong to Brachypodium tribe Poaceae family which has a close phylogentic relationships with the major cereal crops, including wheat, barley and rye. It has many favorable features, such as small genome (~300 Mb), diploid accessions, self-fertility, a short lifecycle and easy transformation, which make it an ideal model organism for functional genomic studies of temperate grasses, cereals and biofuel crops [23, 24] and now its genome has been completely sequenced [25]. The available genome data facilitated the studies to reveal the gene function and regulation network in this species, and the study of *B. distachyon* will provide the vital clue for better understand the molecular mechanism of stress response and subsequently improve the abiotic stress tolerance of other cereal crops. So far, the AP2/ERF family has been identified in Arabidopsis [1], Bamboo [26], grapevine [27], maize [28], peach [29] and rice [30]. However, to the best of our knowledge, the systematic identification of AP2/ERF family has not been performed in *B. distachyon*, limiting the further function analysis of this important gene family.

In this study, a genome-wide bioinformatics analysis was conducted to investigate the genomic organization, phylogenetic relationship and expression profiles of AP2/ERF genes in *B. distachyon*. The chromosomal localization, gene structures, cis-elements in the promoter region as well as gene duplication and evolutionary mechanisms were subsequently analyzed. By using RNA-seq and microarray expression data, the expression profiling of these identified AP2/ERF genes in different tissue as well as under cold and drought stresses was further investigated. Our study provided a basis for further study on the regulation roles of the AP2/ERF family playing in *B. distachyon* development and in response to biotic and abiotic stresses, which will not only provide the helpful information on the evolutionary mechanism of this TFs family in plant, but also contribute to revealing the molecular mechanism of development and stresses response in *B. distachyon* and other cereal crops.

## Methods

### Identification of AP2/ERF gene family in Brachpodium genome

The whole genome data of *B. distachyon* was available at Ensemble plants database (http://plants.ensembl.org/index.html). The predicted protein sequences were downloaded as the dataset for downstream analysis (v1.0.29). The AP2/ERF domain (PF00847) obtained from PFAM database (http://pfam.xfam.org/) was used as the query for Hidden Markov Model (HMM) search using HMMER 3.0 program with a pre-defined threshold of E <1e^−5^. Furhtermore, the AP2/ERF protein sequences ofArabidopsis and rice were obtained from the plant transcription factor database (http://plntfdb.bio.uni-potsdam.de/v3.0/) and then used as query to search against the Brachpodium protein dataset using the BLASTP program with an e-value of 1e-5 and identity of 50 % as the threshold. Furthermore, HMMER and BLAST hits were compared and parsed and then a self-blast of these sequences was performed to remove the redundancy and no any alternative splice variants were considered. After manual correcting, the putative BdAR2/ERF proteins were obtained. Then, the NCBI-CDD web server (http://www.ncbi.nlm.nih.gov/Structure/cdd/wrpsb.cgi) and SMART database (http://smart.embl-heidelberg.de/webcite) were used to further confirm the predicted BdAR2/ERF genes. The theoretical isoelectric point (PI) and molecular weight (MW) of the obtained proteins were conducted by the compute pI/Mw tool in the ExPASy server (http://www.expasy.org/). The subcellular localization prediction of each gene was predicted using the cello web server (http://cello.life.nctu.edu.tw/).

### Multiple sequence alignment and phylogenetic analysis

Multiple sequence alignment was performed using Clustal X v2.0 [31] with the default parameters. An un-rooted neighbor joining (NJ) tree with 1000 bootstrap replications was constructed using MEGA 6.0 [32] based on the full-length protein alignment.

### Chromosome distribution, gene structure and conserved motif analysis

The chromosome distribution of these genes were obtained from the genome annotation information, and then validated by BLASTN search. The exon-intron organizations and splicing phase of these predicted AR2/ERF genes were also investigated based on the annotation file of *B. distachyon* genome, and then graphically displayed by the Gene Structure Display Server (http://gsds.cbi.pku.edu.cn/). Conserved motifs or domains were predicted using the MEME Suite web server (http://meme-suite.org/), with the following parameters: maximum number of motifs set at 25 and optimum with of motifs set from 5 to 200 amino acids.

### Promoter analysis and identification of miRNAs targets

The upstream 2 kb genomic DNA sequences of each predicted AR2/ERF genes were extracted from the *B. distachyon* genome, and then submitted to PLACE database (http://www.dna.affrc.go. jp/PLACE/) to identify the putative cis-regulatory elements in the promoter regions. Furthermore, all the identified AP2/ERF transcripts were searched against the published *B. distachyon* miRNAs in the miRBase using psRNATarget tool (http://plantgrn.noble.org/psRNATarget/) to predict the AR2/ERF targeted by miRNA.

### Gene duplication and synteny analysis

Gene duplication events were identified manually using the method as described by Chen et al. [33]. The segmental duplication events were characterized as copying the whole blocks of genes from one chromosome to another, while contiguous homologous genes with the original duplication on a single chromosome were defined as tandem duplications [34]. For synteny analysis, duplications between *B. distachyon* AP2/ERF genes, as well as the synteny block of this family among *B. distachyon* and other 5 grass species (rice, maize, sorghum, foxtail millet and switchgrass) were obtained from the Plant Genome Duplication Database (http://chibba.pgml.uga.edu/duplication/) and the diagrams were visualized using the program Circos v0.67 [35].

### Gene expression and network interaction analysis

Microarray data of *B. distachyon* were obtained from Gene Expression Omnibus (GEO) (http://www.ncbi.nlm.nih.gov/geo/) and EBI ArrayExpress (https://www.ebi.ac.uk/arrayexpress/) databases, and then used to detect the expression of the AR2/ERFs in different tissue and in response to abiotic stresses. Additionally, high throughput RNA sequencing data were also retrieved and downloaded from the SRA database (http://www.ncbi.nlm.nih.gov/sra) and then used to detect the differential expression of the AR2/ERF genes by FPKM analysis. A total of 9 RNA data of different tissues at different development stages were used, including anther, pistil, leaves (20 days), seed (5 and 10 days after pollination), endosperm(25 days after pollination), embryo(25 days after pollination), and inflorescence (early and emerging time). Finally, the interaction network which these putative AR2/EFR genes involved in were investigated based on the orthogous genes between *B. distachyon* and Arabidopsis using the AraNet V2 tool (http://www.inetbio.org/aranet/) [36].

### Plant growth, stress treatment and RT-PCR analysis

Roots, stems, leaves and spikes were collected from two-months-old Bd21 genotype for RNA extraction and then used for organ-specific expression analysis. The 3 weeks old seedling were subjected to 4 °C, 20 % PEG, 150 mM NaCl conditions as cold,drought and salt treatments. After 24 hours treatment, the leaves of plant under these 3 stresses were collected for RNA isolation, respectively. Total RNA was isolated using RNAiso Reagent (TaKaRa, Dalian, China) according to the manufacturer’s instructions. Semi-quantitative RT-PCR was employed to determine the transcript levels of 11 randomly selected BdAP2/ERF genes following the method as described by Chen et al. [33]. The primers are listed in Additional file 1: Table S1.

## Results

### Identification of AP2/ERF family in Brachypodium

Using the method as described above, a total of 141 genes were identified as putative AP2/ERF genes in the Brachypodium genome, accounting for approximately 0.45 % of all annotated Brachypodium genes. Previous study has reported there were 146 AP2/ERF genes in Brachypodium through exploration of genes encoding TF domains to construct TF database [37]. The difference between them were further compared and results found that previous study considered the alternative splices transcripts encoded by the same gene into different AP2/ERF members, which resulted in the increase of the gene number. Since there is no standard nomenclature, the predicted BdAP2/ERF genes were then designated as BdAP2/ERF001 to BdAP2/ERF141 based on their chromosome location and family classification (Table 1). The detailed sequence information including genomic, transcript, CDS and protein sequence as well as 2 kb upstream has been listed in Additional file 2. Among them, 24 genes containing two repeated AP2/ERF domains were assigned to the AP2 family, and 4 genes possessed a single AP2/ERF DNA binding motif together with a B3 type domain were grouped into the RAV family. The remaining 113 genes with a single AP2/ERF domain were assigned to the ERF superfamily and further divided into ERF and DREB subfamilies. Additionally, a special AP2/ERF gene, namely BdAP2/ERF091 showed little similarity to other AP2/ERF genes, which was grouped into Soloist subfamily (Table 2 and Additional file 1: Table S2).

**Table 1:** Characteristic features of AP2/ERF Transcription factor gene family identified in *B. distachyon*

Gene Name	Ggene id	Physical position			Properties of AP2/ERF proteins			Subcell location	EST valadation
		Chrom no	Start position (bp)	End Position (bp)	Protein length (aa)	pI	Molecular weight (Da)		
BdAP2/ERF001	Bradi1g00670	1	521659	522548	192	8.13	20.2498	Nuclear	17
BdAP2/ERF002	Bradi1g03880	1	2601482	2604919	451	7.23	48.97473	Nuclear	6
BdAP2/ERF003	Bradi1g04110	1	2788453	2789893	302	10.05	32.70946	Nuclear	31
BdAP2/ERF004	Bradi1g07290	1	5105505	5109153	635	6.54	67.00742	Nuclear	-
BdAP2/ERF005	Bradi1g18580	1	14895085	14896008	308	6.13	32.25646	Nuclear	8
BdAP2/ERF006	Bradi1g18870	1	15104782	15107266	260	9.9	27.76687	Mitochondrial	-
BdAP2/ERF007	Bradi1g23756	1	19130000	19131273	299	9.05	32.04507	Nuclear	5
BdAP2/ERF008	Bradi1g30337	1	25719025	25721056	379	9.07	40.85655	Nuclear	1
BdAP2/ERF009	Bradi1g31337	1	26832687	26835421	467	5.81	50.91159	Nuclear	2
BdAP2/ERF010	Bradi1g33550	1	29119163	29120322	189	5.17	20.18015	Nuclear	2
BdAP2/ERF011	Bradi1g35400	1	30927335	30927973	213	9.16	22.94622	Chloroplast	-
BdAP2/ERF012	Bradi1g35410	1	30934707	30935330	208	8.33	22.09309	Nuclear	-
BdAP2/ERF013	Bradi1g35420	1	30939140	30939700	187	5.11	20.32711	Cytoplasmic	-
BdAP2/ERF014	Bradi1g36590	1	32262325	32263730	226	9.3	23.87795	Nuclear	-
BdAP2/ERF015	Bradi1g38110	1	34253209	34254003	265	4.85	27.79697	Chloroplast	1
BdAP2/ERF016	Bradi1g45470	1	43685130	43686821	352	7.79	37.90218	Nuclear	7
BdAP2/ERF017	Bradi1g46120	1	44406118	44407133	236	4.62	24.42913	Chloroplast	2
BdAP2/ERF018	Bradi1g46690	1	45270114	45272465	352	4.78	38.51971	Nuclear	53
BdAP2/ERF019	Bradi1g47480	1	46027554	46028362	154	6.97	16.7786	Nuclear	5
BdAP2/ERF020	Bradi1g48320	1	46956726	46957256	177	9.99	18.74815	Nuclear	23
BdAP2/ERF021	Bradi1g49560	1	48258020	48259205	221	5.32	23.59387	Nuclear	5
BdAP2/ERF022	Bradi1g49570	1	48261395	48262309	225	5.62	23.88046	Nuclear	8
BdAP2/ERF023	Bradi1g53650	1	51957465	51961449	415	6.14	45.29458	Nuclear	9
BdAP2/ERF024	Bradi1g54450	1	52809017	52809787	257	9.32	27.56207	Nuclear	3
BdAP2/ERF025	Bradi1g57560	1	56305875	56308647	605	7.17	63.09426	Nuclear	-
BdAP2/ERF026	Bradi1g57970	1	56789254	56789982	243	4.7	26.05227	Chloroplast	9
BdAP2/ERF027	Bradi1g64240	1	63443708	63448081	395	9.09	42.75122	Nuclear	-
BdAP2/ERF028	Bradi1g67350	1	65990605	65991651	247	5.25	25.45989	Nuclear	4
BdAP2/ERF029	Bradi1g69207	1	67693295	67697013	628	6.98	67.31695	Nuclear	2
BdAP2/ERF030	Bradi1g71740	1	69679681	69681013	288	6.17	31.22614	Nuclear	7
BdAP2/ERF031	Bradi1g72450	1	70182711	70183727	339	4.78	35.80922	Nuclear	63
BdAP2/ERF032	Bradi1g72457	1	70186902	70188241	308	5.82	32.56157	Chloroplast	10
BdAP2/ERF033	Bradi1g72890	1	70503793	70507456	526	7.82	55.3123	Nuclear	10
BdAP2/ERF034	Bradi1g72990	1	70580747	70581774	331	6.41	34.74665	Nuclear	4
BdAP2/ERF035	Bradi1g75040	1	72026796	72027191	132	6.61	14.16364	Nuclear	7
BdAP2/ERF036	Bradi1g77120	1	73494001	73494771	257	5.43	27.522	Chloroplast	8
BdAP2/ERF037	Bradi2g02100	2	1434535	1436793	337	4.77	36.11855	Nuclear	14
BdAP2/ERF038	Bradi2g02710	2	1905801	1907344	364	7.16	39.28463	Nuclear	17
BdAP2/ERF039	Bradi2g02720	2	1921269	1922752	365	9.96	38.9569	Chloroplast	14
BdAP2/ERF040	Bradi2g04000	2	2818870	2820722	280	5.97	30.81911	Nuclear	1
BdAP2/ERF041	Bradi2g06180	2	4632942	4633697	252	4.8	26.7444	Nuclear	-
BdAP2/ERF042	Bradi2g07357	2	5714188	5715258	357	4.85	38.60973	Nuclear	9
BdAP2/ERF043	Bradi2g09434	2	7714796	7719951	1338	5.98	148.56946	Nuclear	2
BdAP2/ERF044	Bradi2g11890	2	10206158	10207184	198	6.19	21.45306	Nuclear	57
BdAP2/ERF045	Bradi2g15847	2	14025073	14025927	285	9.51	30.25695	Nuclear	7
BdAP2/ERF046	Bradi2g17610	2	15668356	15669979	409	9.64	43.71991	Nuclear	29
BdAP2/ERF047	Bradi2g18570	2	16501499	16503701	454	8.62	48.82772	Chloroplast	-
BdAP2/ERF048	Bradi2g21060	2	18434490	18435619	237	8.87	23.84977	Nuclear	19
BdAP2/ERF049	Bradi2g21067	2	18444903	18445651	196	10.25	20.59425	Nuclear	4
BdAP2/ERF050	Bradi2g24170	2	22011630	22012545	228	6.51	23.49464	Nuclear	15
BdAP2/ERF051	Bradi2g25050	2	22846421	22847117	180	9.63	19.10829	Nuclear	-
BdAP2/ERF052	Bradi2g26987	2	25743726	25748213	394	7.6	43.00918	Chloroplast	-
BdAP2/ERF053	Bradi2g27920	2	26951955	26953071	169	6.63	17.67988	Chloroplast	10
BdAP2/ERF054	Bradi2g29960	2	29508803	29511680	382	5	41.65054	Nuclear	15
BdAP2/ERF055	Bradi2g31480	2	31231590	31233055	272	8.54	29.35912	Nuclear	5
BdAP2/ERF056	Bradi2g37800	2	38179562	38184091	494	6.48	53.7679	Nuclear	12
BdAP2/ERF057	Bradi2g45530	2	45915509	45916627	301	4.44	31.9448	Nuclear	9
BdAP2/ERF058	Bradi2g47220	2	47558425	47559938	404	9.4	42.57902	Chloroplast	32
BdAP2/ERF059	Bradi2g48130	2	48444901	48447985	349	9.12	39.41296	Mitochondrial	-
BdAP2/ERF060	Bradi2g52370	2	51766250	51767401	244	9.57	25.15542	Nuclear	9
BdAP2/ERF061	Bradi2g52380	2	51772580	51773238	172	10.25	18.47806	Nuclear	-
BdAP2/ERF062	Bradi2g53070	2	52290848	52293688	436	8.91	47.4695	Nuclear	-
BdAP2/ERF063	Bradi2g56140	2	54552519	54554079	252	7.28	26.8432	Nuclear	19
BdAP2/ERF064	Bradi2g57200	2	55409637	55410303	147	6.61	15.52733	Nuclear	6
BdAP2/ERF065	Bradi2g57747	2	55829067	55832804	628	6.17	66.83063	Nuclear	1
BdAP2/ERF066	Bradi2g60331	2	57699689	57700420	244	5.18	25.33294	PlasmaMembrane	18
BdAP2/ERF067	Bradi2g60340	2	57707368	57708090	241	4.9	25.10965	Chloroplast	21
BdAP2/ERF068	Bradi2g61630	2	58640558	58642834	331	11.63	35.95634	Nuclear	17
BdAP2/ERF069	Bradi3g04370	3	2981846	2982121	92	10.92	9.77404	Nuclear	-
BdAP2/ERF070	Bradi3g04380	3	2988281	2989361	118	9.69	12.80229	Nuclear	-
BdAP2/ERF071	Bradi3g04410	3	3030811	3031468	194	6.58	19.75096	Nuclear	9
BdAP2/ERF072	Bradi3g06562	3	4743747	4744779	195	5.32	20.50215	Nuclear	3
BdAP2/ERF073	Bradi3g07450	3	5592374	5593517	212	9.07	22.84071	Nuclear	20
BdAP2/ERF074	Bradi3g08790	3	6915336	6916085	250	5.5	26.42149	Nuclear	2
BdAP2/ERF075	Bradi3g12565	3	11243949	11244320	124	8.85	13.81715	Nuclear	14
BdAP2/ERF076	Bradi3g12680	3	11374939	11375580	214	8.74	22.85931	Nuclear	-
BdAP2/ERF077	Bradi3g15880	3	14106918	14109546	288	5.41	31.29551	Nuclear	2
BdAP2/ERF078	Bradi3g18070	3	16466074	16466826	171	10.58	17.59787	Nuclear	8
BdAP2/ERF079	Bradi3g24000	3	23546847	23547533	229	8.73	24.80911	Nuclear	4
BdAP2/ERF080	Bradi3g27690	3	28745318	28745683	122	5.57	12.79902	Cytoplasmic	2
BdAP2/ERF081	Bradi3g31600	3	33875837	33876445	203	5.26	21.76436	Chloroplast	8
BdAP2/ERF082	Bradi3g33355	3	35731449	35732636	264	4.9	27.94319	Nuclear	3
BdAP2/ERF083	Bradi3g33670	3	36046660	36047028	123	10.08	13.23904	Nuclear	-
BdAP2/ERF084	Bradi3g35560	3	37854781	37856423	275	9.46	29.16776	Nuclear	38
BdAP2/ERF085	Bradi3g36820	3	39192052	39195892	376	7.09	41.41683	Nuclear	-
BdAP2/ERF086	Bradi3g37544	3	40042914	40043558	215	4.89	22.74426	Nuclear	-
BdAP2/ERF087	Bradi3g38140	3	40608825	40610019	280	5.69	30.34868	Nuclear	24
BdAP2/ERF088	Bradi3g41543	3	43477691	43479816	236	6.71	25.05387	Nuclear	4
BdAP2/ERF089	Bradi3g41546	3	43481400	43482857	234	8.44	24.97712	Chloroplast	-
BdAP2/ERF090	Bradi3g42627	3	44115159	44119544	487	8.75	53.34822	Chloroplast	-
BdAP2/ERF091	Bradi3g43822	3	45506144	45512571	276	9.2	31.0089	Nuclear	21
BdAP2/ERF092	Bradi3g44470	3	46345345	46346091	249	5.07	26.56773	Nuclear	13
BdAP2/ERF093	Bradi3g45997	3	47939264	47939779	172	5.2	18.7198	Nuclear	-
BdAP2/ERF094	Bradi3g47610	3	49227063	49228217	308	6.97	32.78146	Nuclear	1
BdAP2/ERF095	Bradi3g48697	3	50080298	50084464	690	6.11	73.30758	Nuclear	-
BdAP2/ERF096	Bradi3g49810	3	51052812	51054767	437	5.72	47.32396	Nuclear	22
BdAP2/ERF097	Bradi3g50490	3	51687040	51688418	297	6.32	30.96065	Nuclear	24
BdAP2/ERF098	Bradi3g50620	3	51763593	51764381	263	5.44	27.54566	Nuclear	2
BdAP2/ERF099	Bradi3g50630	3	51784618	51785497	238	5.35	24.99462	Nuclear	7
BdAP2/ERF100	Bradi3g51610	3	52662982	52664420	286	4.77	29.4364	Nuclear	-
BdAP2/ERF101	Bradi3g51630	3	52685121	52686098	228	5.87	24.17896	Nuclear	5
BdAP2/ERF102	Bradi3g54160	3	54676859	54677475	169	8.8	18.11231	Nuclear	6
BdAP2/ERF103	Bradi3g57360	3	57003839	57004549	237	5.39	25.75201	Chloroplast	5
BdAP2/ERF104	Bradi3g57867	3	57514740	57522455	545	6.05	58.32911	Chloroplast	49
BdAP2/ERF105	Bradi3g58015	3	57607422	57608473	259	5.54	27.34452	Extracellular	9
BdAP2/ERF106	Bradi3g58980	3	58281872	58283733	316	7.02	33.64749	Nuclear	33
BdAP2/ERF107	Bradi3g59300	3	58524999	58529801	373	5.87	40.81537	Nuclear	8
BdAP2/ERF108	Bradi3g60120	3	59149405	59152513	307	4.76	33.5793	Chloroplast	10
BdAP2/ERF109	Bradi4g21265	4	24609196	24609797	193	5	20.80231	Cytoplasmic	-
BdAP2/ERF110	Bradi4g27850	4	33135019	33136560	315	4.61	34.73159	Nuclear	3
BdAP2/ERF111	Bradi4g29010	4	34421980	34423859	283	6.09	30.38067	Nuclear	25
BdAP2/ERF112	Bradi4g30617	4	36372483	36375685	394	7.19	42.82752	Chloroplast	-
BdAP2/ERF113	Bradi4g31040	4	36772794	36775742	402	4.71	43.41933	Nuclear	18
BdAP2/ERF114	Bradi4g35570	4	40939109	40939843	245	5.63	26.34075	Chloroplast	8
BdAP2/ERF115	Bradi4g35580	4	40942477	40943253	259	5.89	27.62901	Nuclear	2
BdAP2/ERF116	Bradi4g35590	4	40947469	40948215	249	4.99	26.7398	Nuclear	9
BdAP2/ERF117	Bradi4g35600	4	40952136	40952876	247	4.72	26.38781	Chloroplast	4
BdAP2/ERF118	Bradi4g35610	4	40959730	40960464	245	5.58	25.92315	Nuclear	6
BdAP2/ERF119	Bradi4g35620	4	40963208	40963969	254	4.94	26.84695	Chloroplast	4
BdAP2/ERF120	Bradi4g35630	4	40965956	40967004	255	5.09	26.88492	Chloroplast	7
BdAP2/ERF121	Bradi4g35650	4	40976869	40977919	239	4.66	25.76346	Nuclear	36
BdAP2/ERF122	Bradi4g38930	4	43634131	43635066	312	6.66	33.31991	Nuclear	14
BdAP2/ERF123	Bradi4g43877	4	47462247	47467299	421	5.46	45.50805	Nuclear	2
BdAP2/ERF124	Bradi5g08380	5	11092852	11094953	286	9.92	30.21775	Nuclear	9
BdAP2/ERF125	Bradi5g14960	5	18387279	18391400	687	5.99	71.21576	Nuclear	-
BdAP2/ERF126	Bradi5g16450	5	19777125	19778588	488	8.34	51.67208	Nuclear	11
BdAP2/ERF127	Bradi5g17480	5	20740745	20742261	290	6.34	30.56629	Nuclear	33
BdAP2/ERF128	Bradi5g17490	5	20752451	20753777	365	5.04	38.57791	Nuclear	1
BdAP2/ERF129	Bradi5g17610	5	20857144	20858031	296	5.4	31.03794	Nuclear	2
BdAP2/ERF130	Bradi5g17620	5	20862925	20863756	252	5.23	26.4537	Chloroplast	3
BdAP2/ERF131	Bradi5g17630	5	20867545	20868171	209	5.96	22.66944	Chloroplast	-
BdAP2/ERF132	Bradi5g17640	5	20872897	20873850	212	5.21	22.43383	Nuclear	-
BdAP2/ERF133	Bradi5g18850	5	21957497	21958102	202	5.22	20.90808	Nuclear	-
BdAP2/ERF134	Bradi5g21250	5	23939710	23940850	217	9.69	22.12031	Nuclear	53
BdAP2/ERF135	Bradi5g24100	5	25842335	25845739	467	6.71	49.18327	Nuclear	2
BdAP2/ERF136	Bradi5g24110	5	25859990	25861523	250	9.55	26.88308	Nuclear	16
BdAP2/ERF137	Bradi5g24360	5	26013512	26017700	522	6.23	55.54319	Nuclear	-
BdAP2/ERF138	Bradi5g24700	5	26290017	26290721	143	6.29	15.30613	Nuclear	-
BdAP2/ERF139	Bradi5g24710	5	26293257	26294078	163	8.89	17.6381	Mitochondrial	-
BdAP2/ERF140	Bradi5g24720	5	26295256	26295857	144	6.09	15.98271	Nuclear	-
BdAP2/ERF141	Bradi5g25570	5	26828492	26829400	188	8.35	19.37177	Nuclear	11

**Table 2:** Summary of the abundance of each group of the AP2/ERF superfamily in *B. distachyon*, Arabidopsis and rice

Family	Subfamily	Group	B. distachyon	Arabidopsis	Rice
AP2			24	18	29
ERF			112	122	139
	DREB		52	57	56
		I	9	10	9
		II	7	16	15
		III	32	22	26
		IV	4	9	6
	ERF		53	65	76
		V	9	12	11
		VI	11	8	6
		VII	7	5	15
		VIII	14	15	13
		IX	8	18	18
		X	6	7	13
	a single group		5		7
RAV			4	6	5
Soloist			1	1	1
Total AP2/ERF genes			141	147	174
genome size (Mbp)			355	125	430
The average number of AP2/ERF family genes per Mb (gene/MB)			0.3972	1.1760	0.4047
The percentage of AP2/ERF family genes (%)			0.45	0.55	0.43

Chromosome distribution analysis found that the BdAP2/ERF genes were unevenly distributed on all of the five chromosomes of Brachypodium. In detail, 40 AP2/ERF genes located on the chromosome 3, representing the most abundant regions, followed by the chromosome 1, 2 and 5, with the number of 36, 32 and 18 respectively, while there were only 15 genes on the chromosome 4, which have the minimum number of AP2/ERFs. Interestingly, all the 4 RAV genes located on the chromosome 2, which may be a Brachypodium-specific feature. The putative proteins of BdAP2/ERFs ranged from 92 to 1338 amino acids in length, with molecular weights (Mw) ranging from 9.8 to 148.6 kDa and theoretical isoelectric points (PI) ranging from 4.33 to 11.63. Subcellular localization analysis indicated that majority of BdAP2/ERFs (108 out of 141, 76.5 %) localized in the nucleus, while 25 genes were predicted to be located in the chloroplast and the remaining 7 genes located in cytoplasmic, mitochondrial, plasma membrane and extra-celluar (Table 1). To further assess the actual existence of these genes identified in this study, all the available Brachypodium expressed sequence tags (EST) were used to search against these genes using the BlastN program. Results showed that most of the AP2/ERFs were supported by EST hits, only 36 genes (25.5 %, 36/111) showed no EST hits. In light of the limit of available ESTs, the not-supported BdAP2/ERF gene might not express under any the used conditions or express with very low level that cannot be detected experimentally.

### Phylogenetic relationship, conserved motif and gene structure analysis

**Fig. 1:**
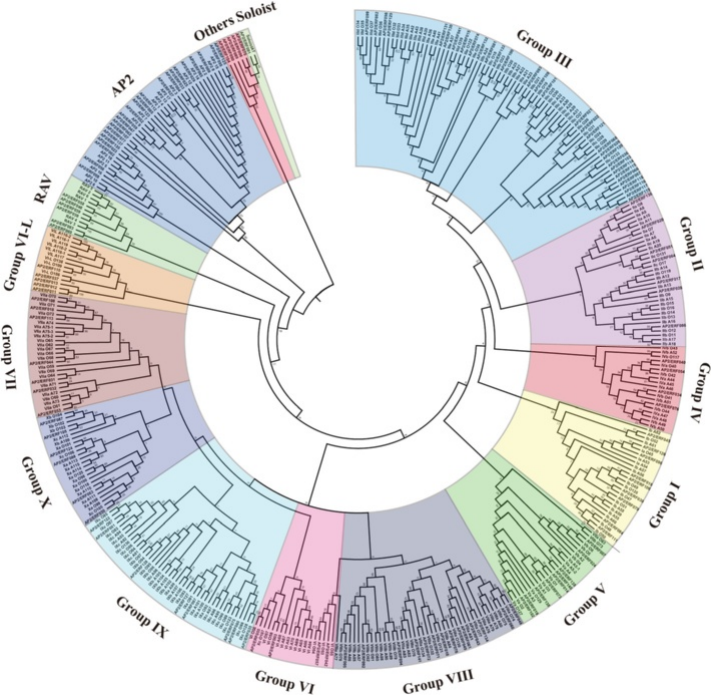
Phylogenetic analysis of AP2/ERF proteins in *B. distachyon*, Arabidopsis and rice. The phylogenetic tree was constructed using the NJ (Neighbor-joining) method with 1000 bootstrap replications

To evaluate the evolutionary relationships of BdAP2/ERF genes, phylogenetic analysis was further conducted based on multiple sequence alignment of all of the BdAP2/ERF together with rice and Arabidopsis AP2/ERF genes. The phylogenetic tree clustered all the AP2/ERF genes into three major clades (ERF, AP2 and RAV) depending on their domain composition as described above (Fig. 1). Furthermore, the ERF clades further divided into ten groups. According to the classification criteria in Arabidopsis and rice [3], the ERF superfamily could be further divided into DREB and ERF subfamily. Four groups (group I-IV) of the ERF clades belonged to ERF subfamily, containing 9, 7, 32 and 4 members while the remaining six groups (V-X) were DREB subfamily, having 9, 11, 7, 14, 8 and 6 members, respectively (Table 2). It’s established that DREB subfamily were major factors involved in plant abiotic stress responses and many stress-inducible DREBs have been isolated from numerous plants to date [21–22, 25,]. The identified DREB genes of *B. distachyon* provided the valuable resource to characterize the stress-responsive genes. Additionally, the bootstrapping values of the nodes in this phylogenetic tree were not very high in every clade, which was consistent with previous studies [3, 38]. NJ-tree reliability was certified by generating another phylogenetic tree by Maximum Parsimony (MP) analysis (Additional file 3: Figure S1), and it was found that nearly all the *BdAP2/ERF* members were placed within the same topological clusters.

**Fig. 2:**
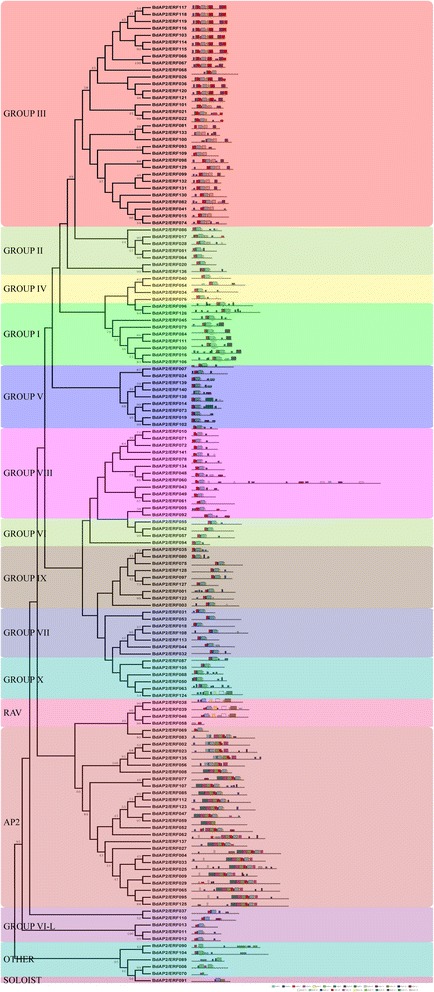
Conserved motifs analysis of BdAP2/ERF genes according to the phylogenetic relationship. Each motif is represented by a number in a colored box. Box length corresponds to motif length

Furthermore, the conserved motifs of BdAP2/ERFs were analyzed and compared. A total of 25 conserved motifs were characterized and named as motif1 to motif25 (Fig. 2 and Additional file 3: Figure S2). Among them, 8 motifs, including motif 1, 2, 3, 4, 6, 7, 16 and 22 were found to be located on the AP2/ERF domain region, while other 17 motifs were corresponded to the regions outside the DNA-binding domain, which was thought to contain either functionally factors, or domains relevant to nuclear localization and transcription regulation [39] (Additional file 1: Table S3). It is noteworthy that proteins within the same group shared one or more motifs that outside the AP2/ERF domain region. For example, motif 19 and 25 were shared by 9 members in the AP2 subfamily. Motifs 12, 15 and 20 were specifically shared by each member in the RAV subfamily, and the motif 11 was shared by ERF group I as well as motif 8, 9, 10, 14 and 18–23 were specifically presented within the group III members in the ERF subfamily. Finally, the motif 24 was shared by the group V in the DREB subfamily. The proteins within the same subfamilies contained the similar composition of conserved motifs, suggesting the similar function may be shared within each group.

**Fig. 3:**
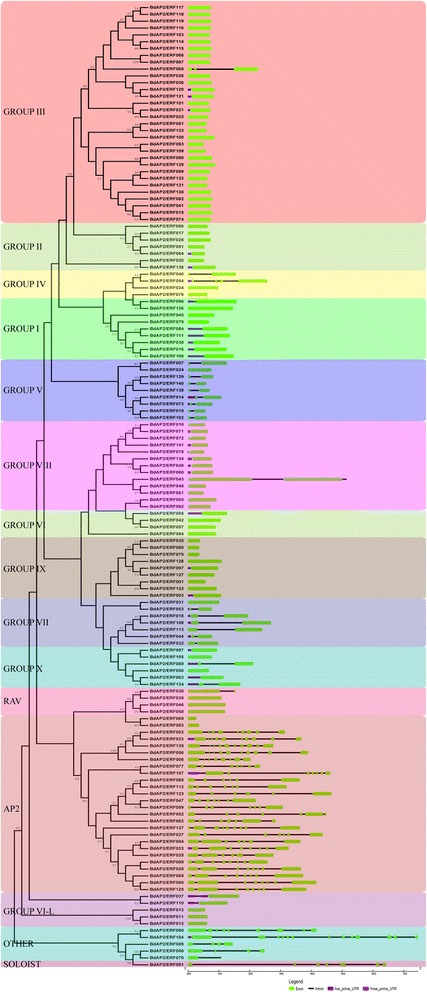
Phylogenetic relationship and gene structure analysis of AP2/ERF genes in Brachypodium

Gene structure analysis of *B. distachyon* AP2/ERF genes further showed that the member within the subfamily possessed the similar exon-intron structures. As a whole, the number of exon regions ranged from 1 to 12, with an average of 2.65. Most of the ERF subfamily genes (74.33 %) were observed to be intronless, which was consistent with the previous study [1]. In contrast, the AP2 subfamily members contained more intron than ERFs, which had at least four exons (Fig. 3). The highly diverse gene structure suggested that vast differentiation may occur during the *B. distachyon* genome formation and evolution.

### Cis-elements and miRNA targets analysis

In order to understand the possible biological functions and regulation network of these AP2/BdERFs involved in, 2 kb genomic sequences upstream of the 5′-UTR of BdAP2/ERF genes were extracted and used to identify cis-regulatory elements. A total of 276 putative cis-elements were found to be presented in at least one BdAP2/ERF gene and only 7 (GT1CONSENSUS, DOFCOREZM, EBOXBNNAPA, MYCCONSENSUSAT, CAATBOX1, CACTFTPPCA1, WRKY71OS) out of them were presented in the promoter region of all BdAP2/ERF genes (Additional file 1: Table S4). In addition, 32 cis-elements were detected as gene-specific, such as S2FSORPL21, ABREDISTBBNNAPA and ABREDISTBBNNAPA were unique to Bradi5g24360, Bradi3g58980 and Bradi5g17620, respectively. The different numbers and types of cis-elements presenting in BdAP2/ERF genes indicated the differential regulatory network which the BdAP2/ERF genes may involve in. Further analysis found that hormones-response (e.g. abscisic acid, gibberellins, auxin, jasmonic acid and ethylene), abiotic stress-related (e.g., drought, extreme temperatures, high salinity, wounding, and disease) and organogenesis-related cis-elements were abundantly presented in the promoter regions of BdAP2/ERF (Additional file 1: Table S5), which indicated that these AP2/ERF genes might have potential functions involving in regulating a variety of stresses response and hormone signaling transduction.

Furthermore, the putative microRNAs (miRNAs) targeted BdAP2/ERF genes were also detected in this study and a total of 8 BdAP2/ERFs were predicted to be targeted by seven miRNAs (Additional file 1: Table S6). Although miRNA inhibition mostly involved the transcript cleavage, the BdAP2/ERF006 was predicted to be inhibited to translation. Most predicted microRNA target sites located into CDS region but outside the AP2 domain, whereas for gene BdAP2/ERF051 the cleavage site located in the 3′UTR region. The miRNAs-AP2/ERF complex identified in this study would be useful in interpreting the post-transcriptional control of gene expression during various stress-induced physiological and cellular processes in *B. distachyon* as well as other cereal crops.

### Gene duplication and synteny analyses of AP2/ERFs between B. distachyon and other three grass species

**Fig. 4:**
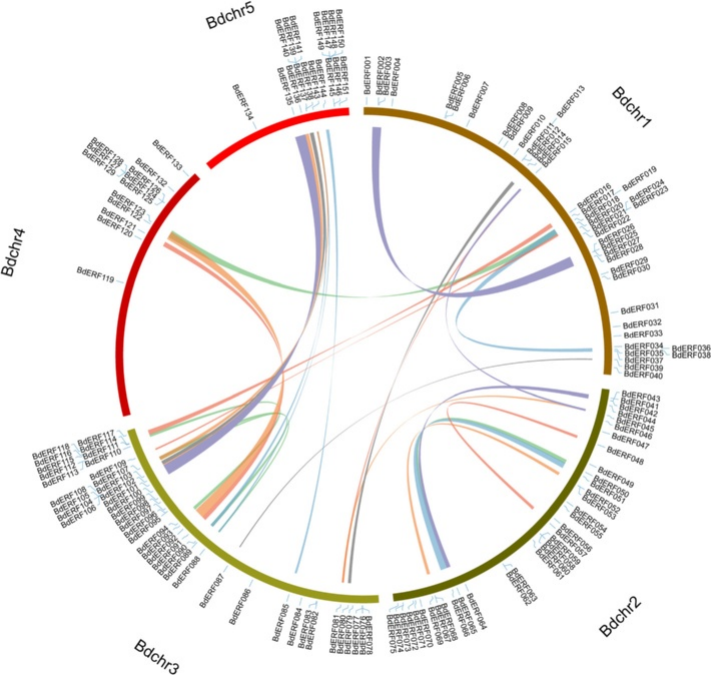
Genomic locations of AP2/EFR genes and duplicated gene pairs in the *B. distachyon* genome

**Fig. 5:**
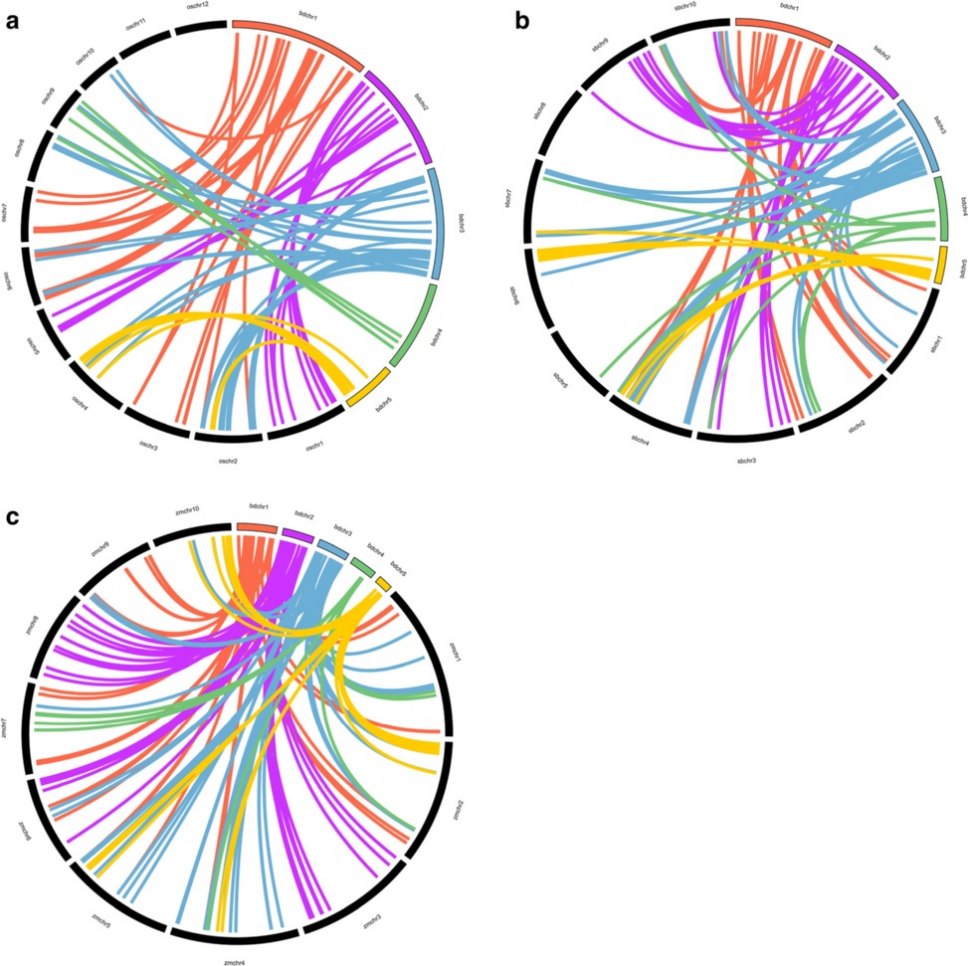
Comparative physical mapping showing the degree of orthologous relationships of BdAP2/ERF genes with (**a**) rice, (**b**) sorghum, (**c**) maize

The tandem and segmental duplication events of BdAP2/ERF genes were investigated through five *B. distachyon* chromosomes (Fig. 4). Four AP2/ERF gene clusters contained twelve tandem duplicated genes were identified, which located on chromosome 1, 2, 4, respectively. Each cluster had a pair of genes except the cluster located on chromosome 4, which contained six genes belonged to group III of ERF subfamily. Furthermore, 27 pairs of chromosomal segments duplication were also found (Fig. 4). Intriguingly, 3 out of 4 RAV family members showed orthologous relationship, suggesting they may share a common ancestor. To derive the origin and evolutionary relationships of AP2/ERF genes, the comparative syntenic analysis between B. distachyon with other three grass species (rice, sorghum and maize) was performed (Fig. 5a, b, c). Through whole genome-wide syntenic analysis, 44, 49 and 48 % of BdAP2/ERF were identified to be orthologous to rice, sorghum and maize, respectively. Most of BdAP2/ERF genes showed syntenic bias towards particular chromosomes of sorghum, maize, rice, which indicated that the chromosomal rearrangement events like duplication and inversion may predominantly shape the distribution and organization of AP2/ERF genes in these genomes.

The substitution rate of non-synonymous (Ka) versus synonymous (Ks) was an effective measure to examine the positive selection pressure after duplication, wherein Ka/Ks =1 means neutral selection, Ka/Ks <1 stands for purifying selection, and Ka/Ks >1 signifies accelerated evolution with positive selection [40]. Furthermore, the divergence rate of the tandem and segmental duplicated BdAP2/ERF genes was calculated to detect selection influence (Additional file 1: Table S7 and S8). The Ka/Ks ratio for tandem duplicated gene-pairs in B. distachyon AP2/ERF genes ranged from 0.23 to 0.51 with an average of 0.31, whereas Ka/Ks for segmental duplicated gene-pairs ranged from 0.19 to 0.85 with an average of 0.53. These results indicated that the duplicated BdAP2/ERF genes were under strong purifying selection pressure and had gone through substitution elimination and enormous selective constraint by natural selection during the process of evolution since their Ka/Ks ratios were estimated to be lower than one. In addition, the duplication event of these BdAP2/ERF tandem and segmental duplicated genes was estimated to have occurred around ~54 and ~61 Mya, respectively. Although the BdAP2/ERF gene-pairs of segmental (Ka/Ks = 0.53) and tandem duplication (Ka/Ks = 0.31) events are not under similar evolutionary positive selection pressure, both set of gene pairs revealed that these duplication events may take place simultaneously. Additionally, the Ka/Ks ratios of the orthologous gene-pairs between B. distachyon and other three grass species were also calculated (Additional file 1: Table S9, S10, S11). The average Ka/Ks value was maximum between *B. distachyo*n and maize (0.47), followed by rice (0.44) and sorghum(0.43), suggesting the genes pairs between *B. distachyon* and those three grass species appeared to have undergone extensive intense purifying selection. The divergence time was about 47, 49 and 51Mya for rice, sorghum and maize, respectively. Therefore, it can be concluded that the segmental and tandem duplication events played a major role in evolution and functional divergent of AP2/ERF genes family in *B. distachyon* as well as other grass species.

### Co-expression network between AP2/ERFs and other genes in B. distachyon

**Fig. 6:**
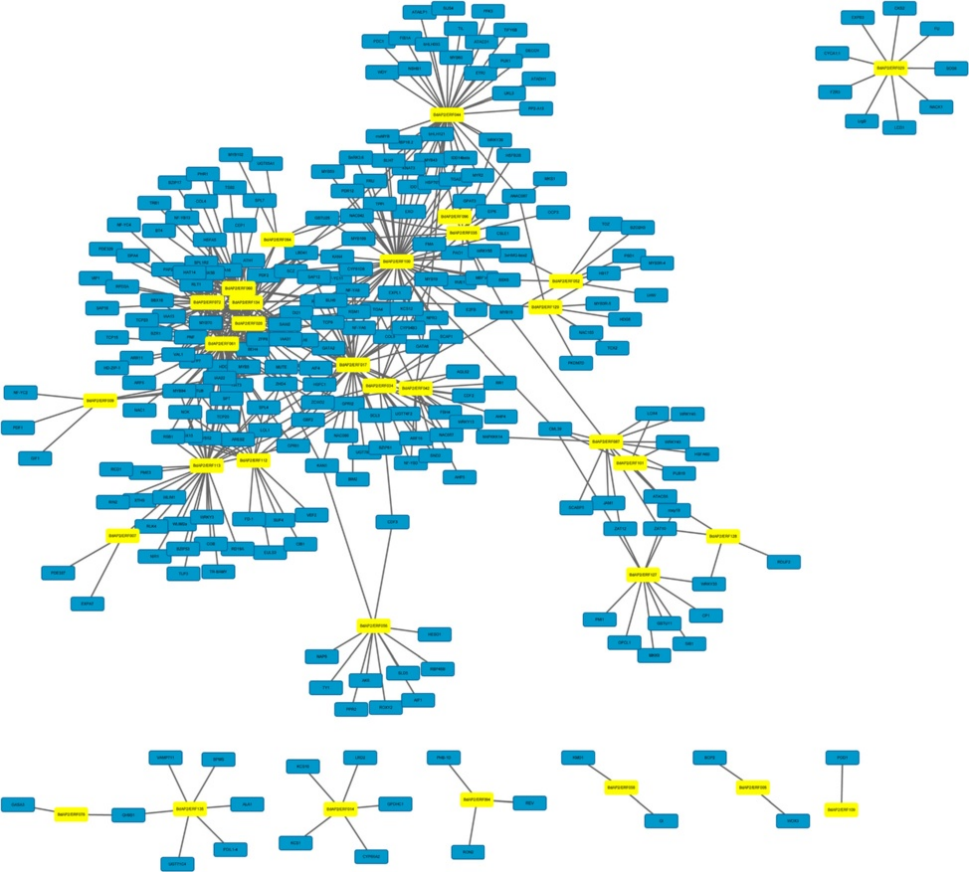
The interaction network of AP2/ERF genes in Brachypdium according to the orthologs in Arabidopsis

To get the preliminary information about the interaction relationship between AP2/ERF and other genes in *B. distachyon*, we constructed the interaction network of them based on the orthology-based prediction followed the network in Arabidopsis (Fig. 6). A total of 39 AP2/ERFs, with 517 gene pairs of network interactions, were detected. The GO annotations of interacted genes were involved diverse biological process, cellular component and molecular function (Additional file 1: Table S12). For example, symbols BLH6, IAA16, IAA31, ZCW32, LBD41 and HAT3, which play an important role in organ development and response to osmotic stress, were identified as the most closed linked genes with AP2/ERFs. Furthermore, we found AP2/ERF61 and AP2/ERF100 regulated 50 downstream genes involved in multiple biological processes, including stress response, hormone, and light response. The co-expression network analysis of AP2/ERF genes may provide important information for the better understanding AP2/ERF transduction pathways in *B. distachyon* as well as in other species.

### Expression profiles of BdAP2/ERF genes at different developmental stages and under stresses

**Fig. 7:**
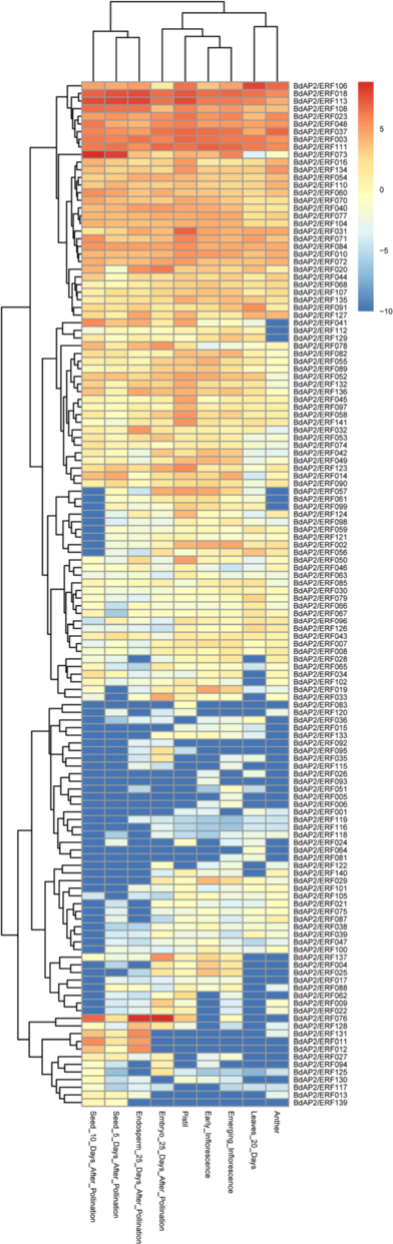
The expression profiles of BdAP2/ERF genes in different tissue and development stage

The tissue-specific expression profiles of BdAP2/ERF genes at different developmental stages were investigated using RNA-Seq data based on the FPKM analysis. Results found there was high variance in the expression levels among BdAP2/ERF genes (Fig. 7). Several proteins showed relatively high expression in all the tissues, including BdAP2/ERF106, BdAP2/ERF018, BdAP2/ERF113, BdAP2/ERF108, BdAP2/ERF023, BdAP2/ERF048, BdAP2/ERF037, BdAP2/ERF003 and BdAP2/ERF111, suggesting they played the indispensable roles in regulating growth and development. However, three genes, including BdAP2/ERF119, BdAP2/ERF116 and BdAP2/ERF118 showed very low expression in all the tested organs. Furthermore, the tissue-specific expressed AP2/EFR genes were also identified. BdAP2/ERF083 and BdAP2/ERF064 were found to be predominantly expressed in pistil and leaf, respectively, while BdAP2/ERF005 and BdAP2/ERF006 showed preferential expression in the emerging inflorescence. In addition, six genes namely BdAP2/ERF092, BdAP2/ERF131, BdAP2/ERF011, BdAP2/ERF012, BdAP2/ERF013 and BdAP2/ERF139 were found to be mainly expressed during pollination, which may contribute to further study of the reproductive growth and seed formation in *B. distachyon*.

**Fig. 8:**
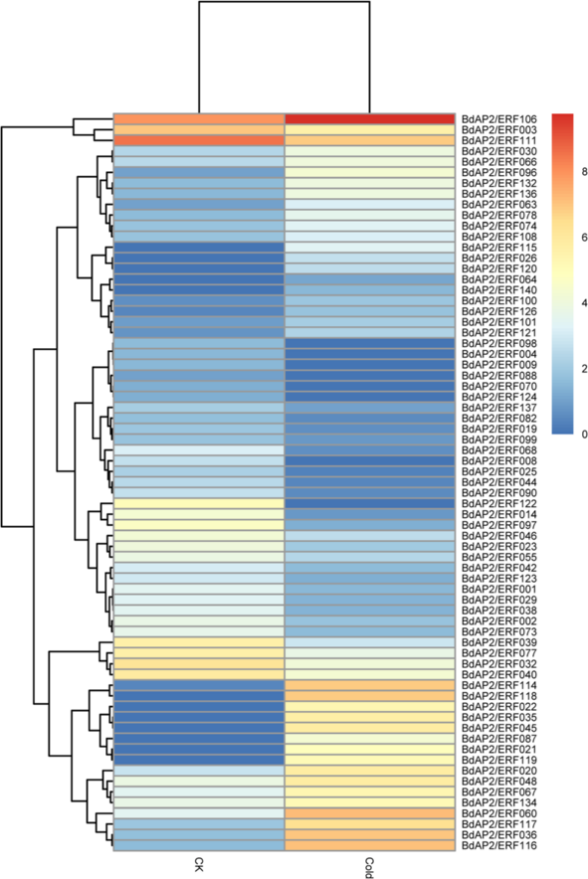
Heatmap of expression profiles of BdAP2/ERF genes under cold stress

**Fig. 9:**
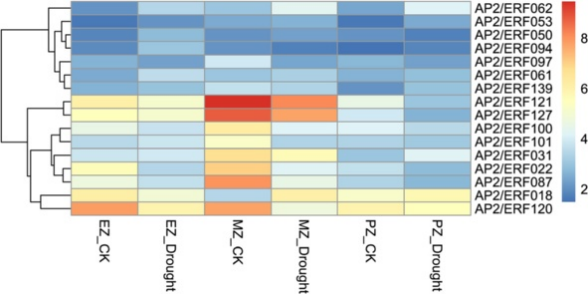
Heatmap of expression profiles of BdAP2/ERF genes under drought stress

To study the roles of BdAP2/ERF genes in the response to abiotic stresses, the RNA-seq data of *B. distachyon* under cold treatment (4 °C, 24 h) [41] was first used to investigate their expression patterns. Based on the RNA-seq data, a total of 106 BdAP2/ERF genes were detected. Using the fold change method (log2-bias ratio) with more than one fold as criterion, 69 genes were identified as differentially expressed genes (Fig. 8). Among them, 34 genes were up-regulated whereas 35 were down-regulated. Remarkably, BdAP2/ERF122 presented 32 fold up-regulated, while BdAP2/ERF118 showed 122 times down-regulated. Furthermore, the expression profiles of BdAP2/ERF genes under drought stress were also analyzed using the available microarray data [42]. Results found that 16 BdAP2/ERF genes were differentially expressed under drought treatment (Fig. 9). In the expansion zone, five genes were identified as differentially expressed genes, of which one was up-regulated, the remaining four was down-regulated. In the mature zone, we detected eight differentially expressed genes, six genes showed up-regulated while the remaining two showed down-regulated. In the proliferation zone, we characterized three up-regulated genes and three down-regulated genes, respectively. Remarkably, AP2/ERF062 showed down-regulated in all three zones, whereas AP2/ERF022 showed up-regulated expansion in zone and mature zone.

### Expression patterns of BdAP2/ERFs in various tissues and under stress treatment by semi-quantitative RT-PCR analysis

**Fig. 10:**
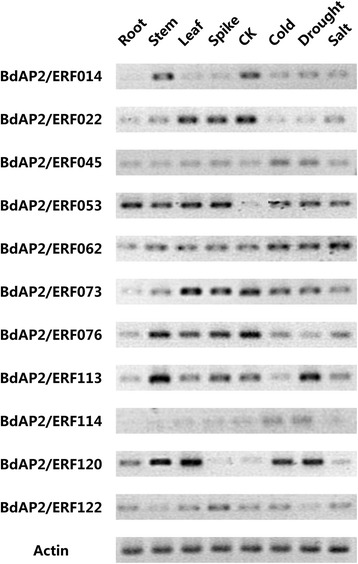
RT-PCR analysis of 11BdAP2/ERF genes

To further verify the expression of these identified AP2/ERF genes, 11 BdAP2/ERF genes were randomly selected to detect their expression levels in four tissues and under three stresses treatments through semi-quantitative RT-PCR analysis (Fig. 10). Results showed only one gene (BdAP2/ERF114) was not expressed in these four tissues and the other ten genes were detected to be expressed. Among them, seven genes were found to be expressed in all four tissues with different profiles. In addition, BdAP2/ERF014 was found to be specifically expressed in stems. BdAP2/ERF076 showed high expression level in stem and leaf, while BdAP2/ERF022 and BdAP2/ERF073 showed high expression level in leaf and spike. Under stress conditions, all of the 11 genes were detected to be expressed. BdAP2/ERF 014, BdAP2/ERF022 and BdAP2/ERF120 were down-regulated under all three stress conditions compared to control, while BdAP2/ERF045, BdAP2/ERF053 and BdAP2/ERF062 was up-regulated under all the treatments. Furthermore, BdAP2/ERF113 showed higher expression under drought treatment, while BdAP2/ERF076 and BdAP2/ERF114 showed high expression under cold and drought treatment respectively, which were consistent with that of RNA-seq and microarray analysis.

## Discussion

AP2/ERF superfamily is one of the largest groups of plant-specific transcription factors, which has been widely studied in diverse plant species, such as Arabidopsis, soybean, rice, maize, foxtail millet and switchgrass [1, 28, 30, 43, 44]. In this study, we performed a comprehensive search for AP2/ERF genes throughout Brachypodium genome, and 141 BdAP2/ERF genes were found, accounting for 0.45 % of all the Brachypodium genes, which was similar with the result in rice (0.43 %), maize (0.44 %) and foxtail millet (0.44 %) [43]. While compared to other plants, the number of AP2/ERF in Brachpodium is much lower than that of rice (174), maize (184) as well as foxtail millet (171), and also slight lower than that of Arabidopsis (148) and grape (149). It has been revealed that the number of AP2/ERF gene family was mainly depending on the number of ERF family members [45]. It’s found that there are 112 members in ERF family in Brachypodium, while 122, 132 and 158 in Arabidopsis, rice and maize, respectively. In contrast, the number of AP2 and RAV family members showed no significantly difference among them, with the value of 28, 24, 34 and 25 in Brachypodium, Arabidopsis, rice and maize respectively. Thus, the lower AP2/ERF gene abundance in Brachpodium may also due to the lower number of ERF and DREB subfamily. Furthermore, the gene density is 0.3972 AP2/ERF genes per Mb in *B. distachyon*, while the value for rice and Arabidopsis is 0.4047 and 1.1760 respectively. *B. distachyon* shows closer AP2/ERF density with rice than Arabidopsis, suggesting the specific evolutionary events might occur to regulate the retention and disposition of this gene family between Monocots and Eudicots.

It has been widely revealed that AP2/ERF transcription factors played crucial roles in regulating plant growth, development and response to diverse stresses as well as signal transduction pathway in plants [46]. However, the function of BdAP2/ERFs is not well understood at present. In this study, the expression patterns of these genes in different tissues and under different stresses were systematically investigated to understand their potential function during development and stress response. Results found that a total of 138 BdAP2/ERFs were expressed in at least one tested tissue, indicating they widely involved in growth and development. Compared to AP2 family, the members in ERF family showed higher expression levels in these tissues. We found that the ERF family genes had less intron than AP2 family in Brachypodium, which may cause the quicker response and higher expression of ERF genes during development [45]. At the same time, BdAP2/ERF genes also showed obvious spatial and temporal expression profiles. For example, BdAP2/ERF064 is specifically expressed in leaf, and BdAP2/ERF005 showed preferential expression in the emerging inflorescence. In addition, six genes having significantly higher expressed during pollination were also identified, which may play the vital role in embryo and endosperm development. AP2/ERF proteins could bind to GCC-box or DRE motifs through the ERF domain, and then regulated the target gene expression under stress conditions [47, 48]. Compare to control, 69 BdAP2/ERFs showed differential expression under cold stress and 16 showed differential expression under drought stress, respectively. Among them, BdAP2/ERF120 (*Bradi4g35630*) which is a member of DREB subfamily, showed significantly up-regulated under both cold and drought stresses. Previous study have reported *Bradi4g35630* encoding a C-repeat binding factor 3-like protein, is a cold-responsive gene and over-expression of this gene could improve the drought, salt and cold tolerance in Brachypodium [49]. Moreover, a total of 5 ACGTATERD1 (element of early responsive to dehydration), 1 DRE1COREZMRAB17 (element of responsive to drought) and 9 MYCCONSENSUSAT (element of responsive to dehydration and cold) cis-elements were identified in the promoter region of BdAP2/ERF120. In addition, BdAP2/ERF053 (Bradi2g27920) is found to be highly expressed in all three stress treatments, which contained 5 LTRECOREATCOR15 (core element of low temperature responsive), 6 EMBP1TAEM (element involving in ABA-mediated stress-signaling pathway) and 1 GT1GMSCAM4 (element required for salt-induced gene expression) cis-elements. We speculated that cis-elements were the vital regulators to control the spatial and temporal expression of the BdAP2/ERFs, which integrated other functional proteins with the AP2/ERF transcription factor to form the complex regulatory metabolic network during development and stress response processes [50]. These identified tissue-specific and stress-induced BdAP2/ERF provided the valuable candidates for further functional studies of AP2/ERF genes in *B. distachyon* as well as in other cereal crops.

## Conclusions

Our current study identified and characterized the AP2/ERF transcription factors in the model grass *B. distachyon*. By performing a genome-wide search, a total of 141 BdAP2/ERF genes were obtained. EST hits or full-length cDNA sequences confirmed their actual existence. The chromosome location, exon-intron structure and conserved motif composition as well as phylogenetic relationship of these BdAP2/ERFs were systematically analyzed and compared. BdAP2/ERFs could be classified into four subgroups in accordance with the number of AP2 domains and putative functions. Co-expression network analysis found that 39 BdAP2/ERFs were involved in regulating other *B. distachyon* genes, and 517 network branches were found. The expression profiles of BdAP2/ERF genes in various tissues as well as under cold and drought stresses were investigated, and several tissue-specific or stress-induced BdAP2/ERF genes were identified, which could considered as the candidates for further study of their function in plant development and stress response. Our study for the first time reported the organization, structure, evolutionary and expression features of the BdAP2/ERF family, which will facilitate the future functional analysis of BdAP2/ERF genes, and lay the foundation for better understanding the molecular mechanism of plant development and stress physiological processes in *B. distachyon* and beyond.

## Abbreviations

AP2/ERF, APETALA2/Ethylene responsive factor; DREB, dehydration responsive element binding protein; EST, expressed sequence tag; FPKM, fragments kilobase of exon model per millon mapped reads; Ka, substitution rate of non-synonymous; Ks, substitution rate of synonymous; MP, maximum parsimony; MW, molecular weight; NJ, neighbor joining; PI, isoelectric point; TF, transcription factors

## Additional files

Additional file 1: Table S1. The primers used in RT-PCR analysis. **Table S2.** Summary of functional domains presented in the BdAP2/ERF proteins. **Table S3.** Detail information of motifs identified in BdAP2/ERF proteins. **Table S4.** Characteristics of cis-regulatory elements in the promoter region of BdAP2/ERF genes. **Table S5.** Annotations of 278 putative cis-acting regulatory DNA elements identified in 141 BdERF genes in *B. distachyon* by PLACE. **Table S6.** List of putative miRNAs targeted BdAP2/ERF predicted by psRNATarget tool. **Table S7.** The Ka/Ks ratios and estimated divergence time for tandemly duplicated BdAP2/ERF genes. **Table S8.** The Ka/Ks ratios and estimated divergence time for segmentally duplicated BdAP2/ERF genes. **Table S9.** The Ka/Ks ratios and estimated divergence time for orthologous BdAP2/ERF proteins between B. distachyon and rice. **Table S10.** The Ka/Ks ratios and estimated divergence time for orthologous BdAP2/ERF proteins between B. distachyon and maize. **Table S11.** The Ka/Ks ratios and estimated divergence time for orthologous BdAP2/ERF proteins between B. distachyon and sorghum. **Table S12.** Detail information of network of BdAP2/ERF with other genes. **Table S13.** Detail information of alternative splicing variants. (XLSX 412 kb) Additional file 2: The detailed sequence information of all the BdAP2/ERF genes, including genomic, transcript, CDS and protein as well as 2 kb upstream sequence. (RAR 245 kb) Additional file 3: Figure S1. A phylogenetic tree of B. distachyon, Arabidopsis and rice AP2/ERF proteins constructed by MP method using MEGA5.0. Then groups are marked I to X. **Figure S2.** Conserved motifs identified from the AP2/ERF genes in *B. distachyon*. (ZIP 3757 kb)
