# Supplementary material for: Genome-wide identification, phylogeny and expression analysis of AP2/ERF transcription factors family in Brachypodium distachyon
Source: BMC Genomics. 2016 Aug 15;17:636. doi: 10.1186/s12864-016-2968-8 (PMC4986339; doi:10.1186/s12864-016-2968-8)
Supplement: Additional file 3: Figure S1. — A phylogenetic tree of B. distachyon, Arabidopsis and rice AP2/ERF proteins constructed by MP method using MEGA5.0. Then groups are marked I to X. Figure S2. Conserved motifs identified from the AP2/ERF genes in B. distachyon. (ZIP 3757 kb) [file 12864_2016_2968_MOESM3_ESM.zip › Figure S2.docx]

**Figure S2. Conserved motifs identified from the AP2/ERF genes in *Brachypodium distachyon***

| MOTIF-1 | 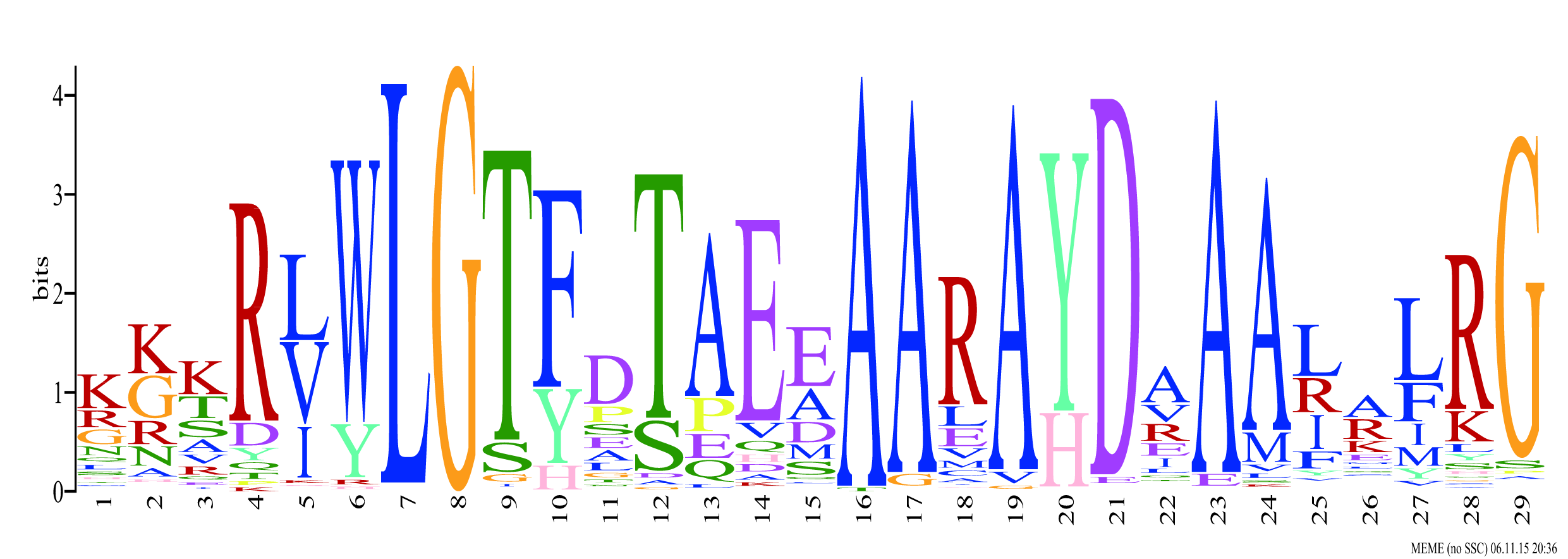 |
| --- | --- |
|  | K[KG]KR[LVI]WLGT[FY]DTAE[EA]AARA[YH]DAAA[LIR][AR][LF]RG |
| MOTIF-2 | 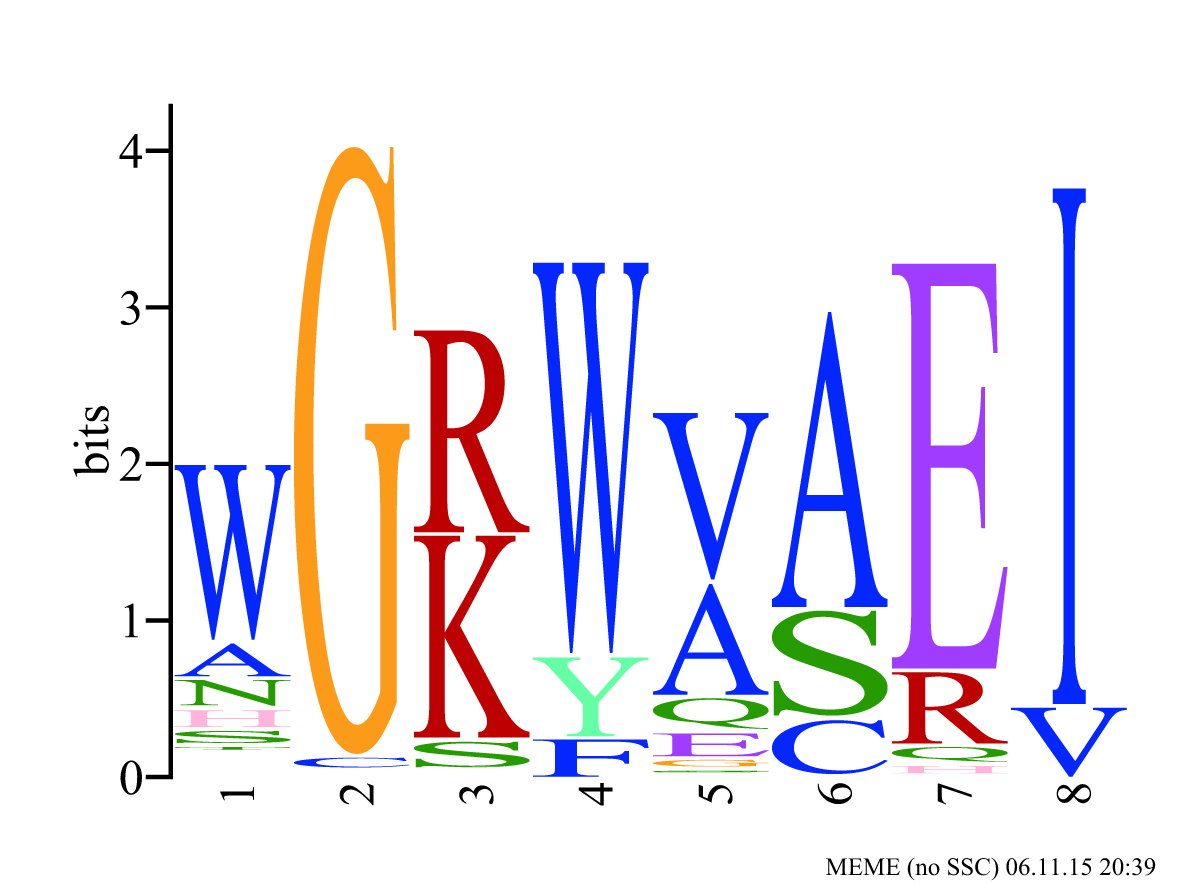 |
|  | WG[KR]W[VA][AS]EI |
| MOTIF-3 | 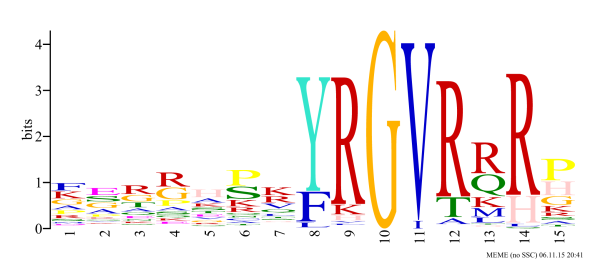 |
|  | xxR[RG]H[PS]K[YF]RGVR[RQ]R[PH] |
| MOTIF-4 | 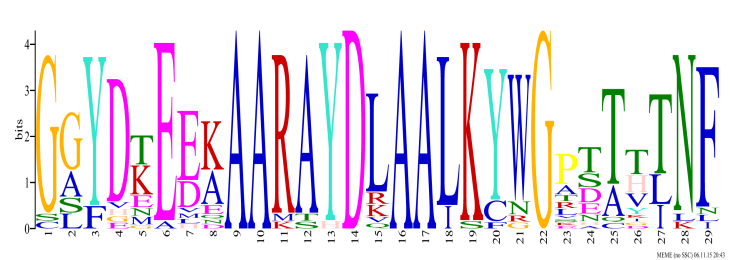 |
|  | G[GA]YD[KT]E[ED][KA]AARAYDLAALKYWGPT[TA][TH][TL]NF |
| MOTIF-5 | 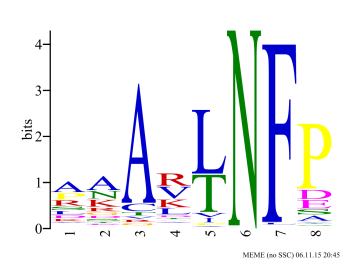 |
|  | AAA[RV][LT]NFP |
| MOTIF-6 | 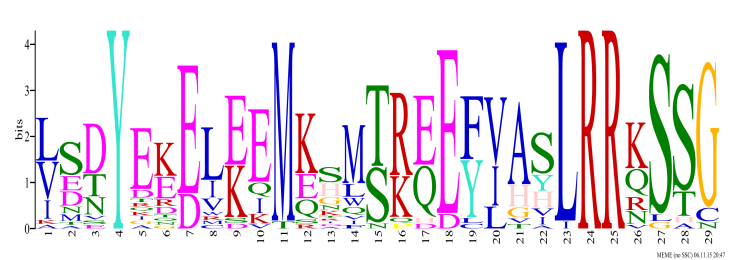 |
|  | [LV]S[DT]YE[KE][ED]L[EK]EM[KE][SH]M[TS][RK][EQ]E[FY][VI]ASLRR[KQ]SSG |
| MOTIF-7 | 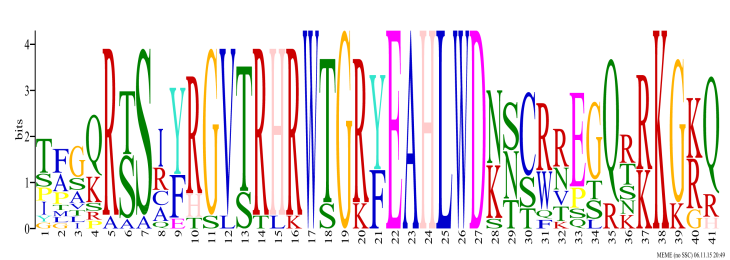 |
|  | T[FA]G[QK]R[ST]S[IR][YF]RGV[TS]RHRWTGR[YF]EAHLWD[NK][NST][CS][RW][RN]EGQ[RT][RK]KG[KR]Q |
| MOTIF-8 | 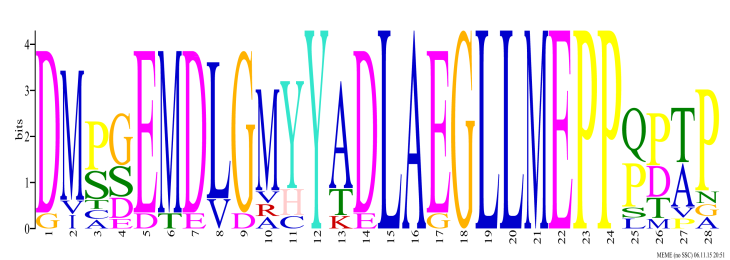 |
|  | DM[PS][GS]EMDLGMYYADLAEGLLMEPP[QP][PD][TA]P |
| MOTIF-9 | 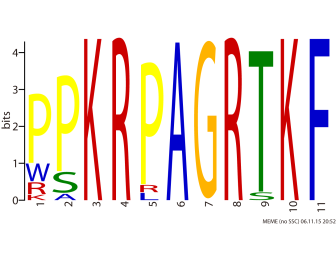 |
|  | PPKRPAGRTKF |
| MOTIF-10 | 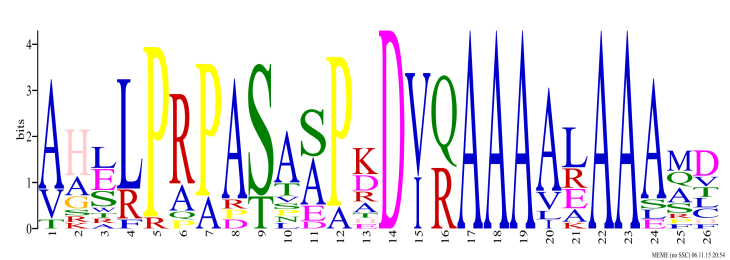 |
|  | [AV][HA][ELS][LR]P[RA][PA]A[ST]A[AS]P[KD]D[VI][QR]AAAA[LER]AAA[MAQ]D |
| MOTIF-11 | 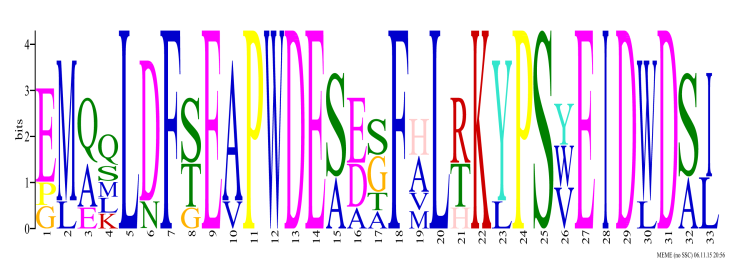 |
|  | EM[QA]QLDF[ST]EAPWDE[SA][ED][GS]F[AH]L[RT]KYPS[VWY]EIDWD[SA][IL] |
| MOTIF-12 | 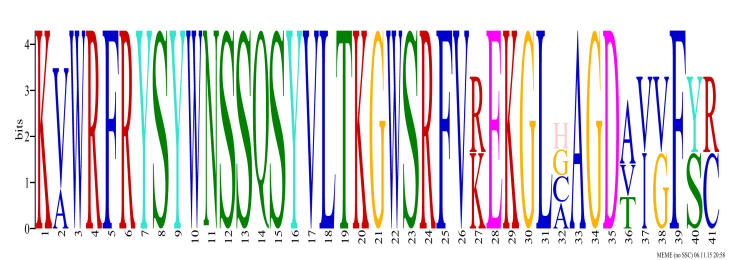 |
|  | K[VA]WRFRYSYWNSSQSYVLTKGWSRFV[KR]EKGL[ACGH]AGD[ATV][IV][GV]F[SY][CR] |
| MOTIF-13 | 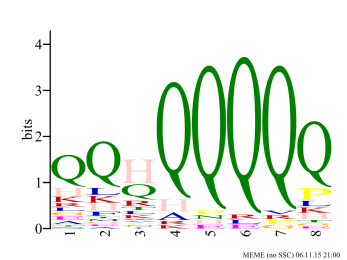 |
|  | QQ[HQ]QQQQQ |
| MOTIF-14 | 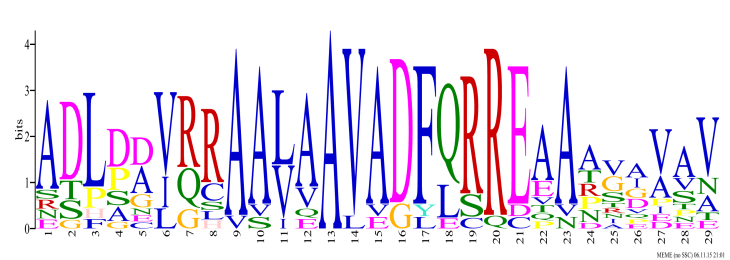 |
|  | ADL[DP][AD][VI][RQ]RAA[LV]AAVADF[QL]RREAAA[GV]A[VA]AV |
| MOTIF-15 | 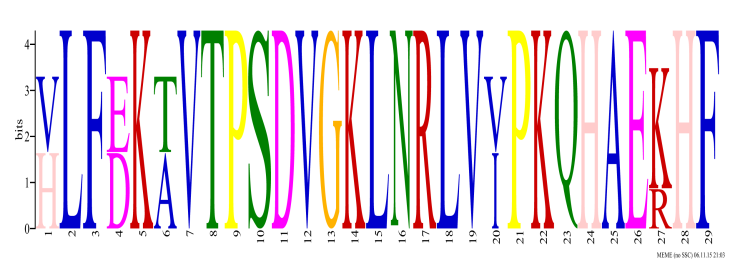 |
|  | [HV]LF[DE]K[AT]VTPSDVGKLNRLV[IV]PKQHAE[KR]HF |
| MOTIF-16 | 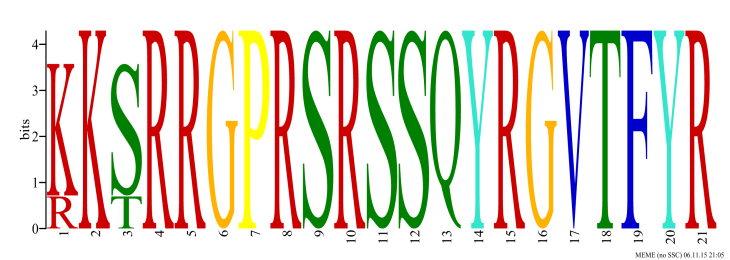 |
|  | [KR]K[ST]RRGPRSRSSQYRGVTFYR |
| MOTIF-17 | 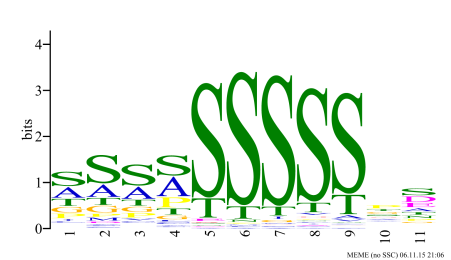 |
|  | [SA]S[SA][SA]SSSSSxx |
| MOTIF-18 | 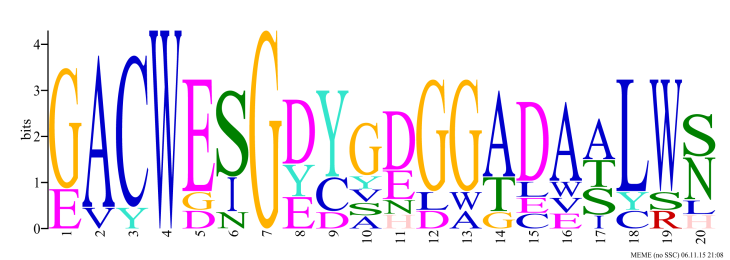 |
|  | [GE]ACWE[SI]G[DEY][YC]G[DE]GG[AT]DA[AST]LW[NS] |
| MOTIF-19 | 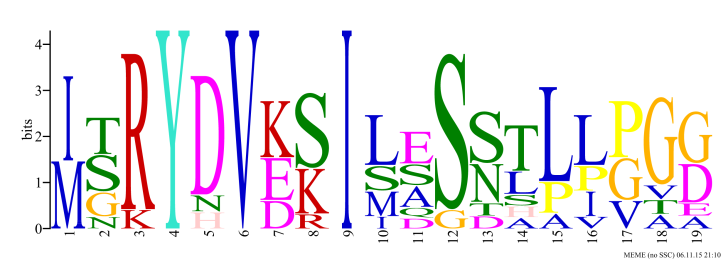 |
|  | [IM][STG]RYDV[KED][SK]I[LMS][EAS]S[SN][TL][LP][LIP][PGV]G[GD] |
| MOTIF-20 | 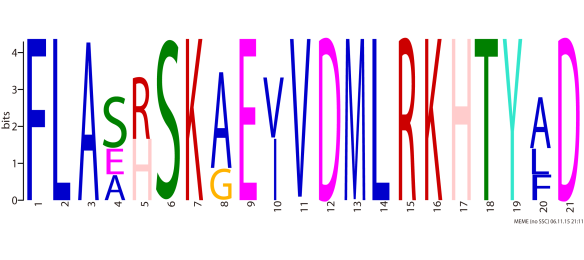 |
|  | FLA[SAE][HR]SK[AG]E[IV]VDMLRKHTY[AFL]D |
| MOTIF-21 | 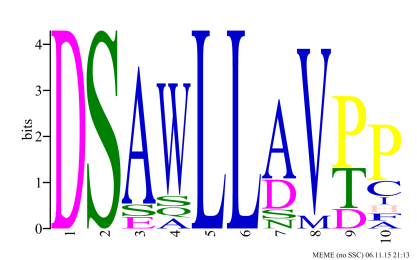 |
|  | DSAWLL[AD]V[PT]P |
| MOTIF-22 | 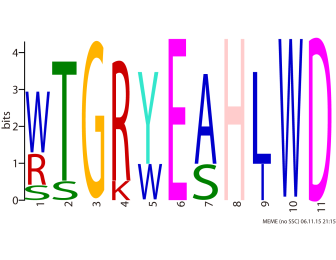 |
|  | [WR]TGR[YW]E[AS]H[LI]WD |
| MOTIF-23 | 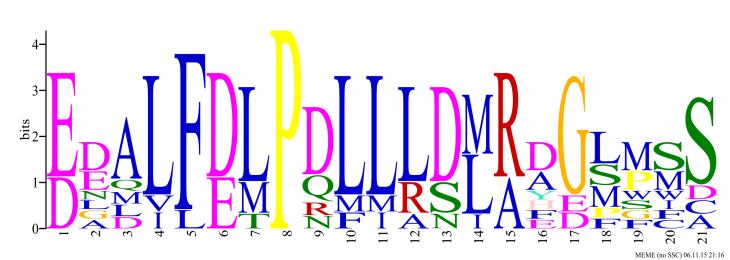 |
|  | [ED][DE]ALF[DE][LM]P[DQ]LL[LR][DS][LM][RA][DA]G[LMS][MP][SM]S |
| MOTIF-24 | 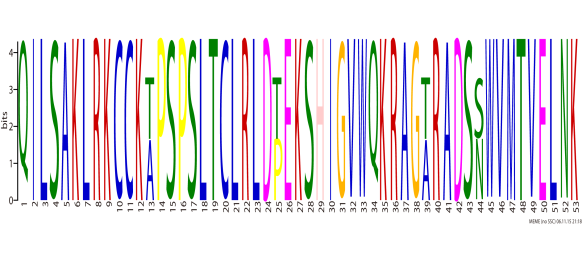 |
|  | QILSAKLRKCCK[AT]PSPSLTCLRLD[PT]EKSHIGVWQKRAG[AT]RADS[NS]WVMTVELNK |
| MOTIF-25 | 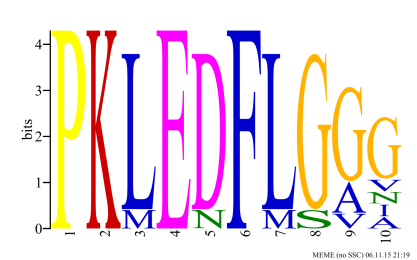 |
|  | PKLEDFLG[GA]G |
