# Supplementary figures and images for: Genome-wide identification, phylogeny and expression analysis of AP2/ERF transcription factors family in Brachypodium distachyon
Source: BMC Genomics. 2016 Aug 15;17:636. doi: 10.1186/s12864-016-2968-8 (PMC4986339; doi:10.1186/s12864-016-2968-8)

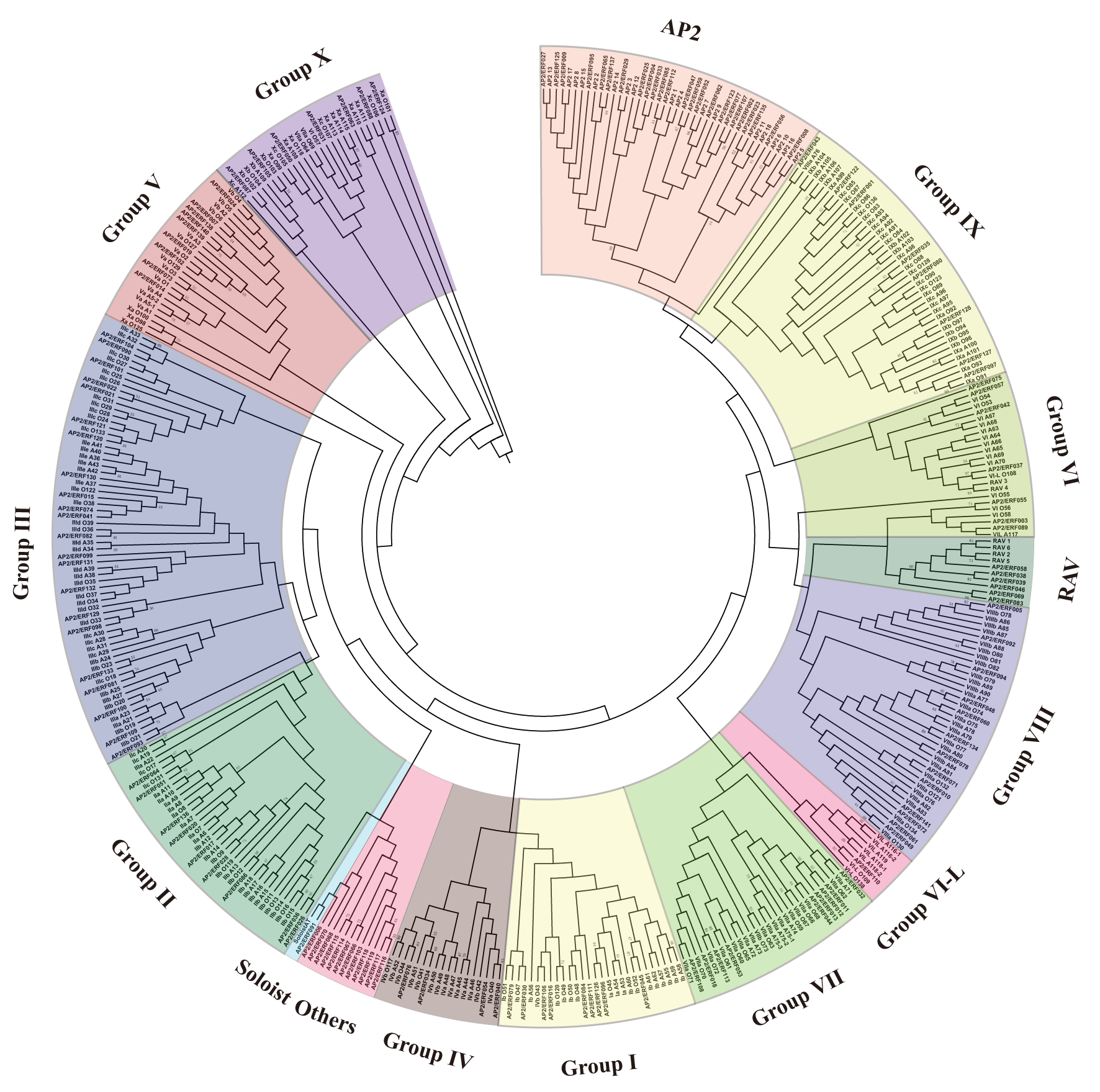

Supplement: Additional file 3: Figure S1. — A phylogenetic tree of B. distachyon, Arabidopsis and rice AP2/ERF proteins constructed by MP method using MEGA5.0. Then groups are marked I to X. Figure S2. Conserved motifs identified from the AP2/ERF genes in B. distachyon. (ZIP 3757 kb) [file 12864_2016_2968_MOESM3_ESM.zip › Figure S1.tif]
